# Changes in Mannitol Content, Regulation of Genes Involved in Mannitol Metabolism, and the Protective Effect of Mannitol on* Volvariella volvacea* at Low Temperature

**DOI:** 10.1155/2019/1493721

**Published:** 2019-06-20

**Authors:** Xu Zhao, Changxia Yu, Yan Zhao, Shunjie Liu, Hong Wang, Chenguang Wang, Ligang Guo, Mingjie Chen

**Affiliations:** ^1^Institute of Edible Fungi, Shanghai Academy of Agricultural Sciences, Shanghai 201403, China; ^2^Institute of Technical Biology & Agriculture Engineering, Hefei Institutes of Physical Science, Chinese Academy of Sciences, Hefei 230031, China; ^3^University of Science and Technology of China, Hefei 230026, China; ^4^Shanghai Baixin Biotechnology Company Limited, Shanghai 201403, China

## Abstract

The mechanism of autolysis of* Volvariella volvacea *(*V. volvacea*) at low temperature has not been fully explained. As mannitol is among the most important osmotic adjustment substances in fungal resistance, this study sampled mycelia of strains V23 and VH3 treated at 0°C for 0, 2, 4, 8, and 10 h to analyze changes in intracellular mannitol content by high-performance anion chromatography with pulsed amperometric detection (HAPEC-PAD). Reverse transcription quantitative PCR (RT-qPCR) analysis was applied to assess differences in the transcript levels of genes associated with mannitol metabolism under low-temperature stress. A mannitol solution was added to cultures of* V. volvacea* fruiting bodies, and effects on the hypothermic resistance of these organs were explored by evaluating variations in sensory properties during cryogenic storage after harvest. The results suggested that in the initial stage of low-temperature treatment, intracellular mannitol was largely catabolized as an energy storage material and the expression of genes encoding enzymes involved in synthetic reactions was inhibited. However, low-temperature resistance was induced with further treatment, with activation of mannitol synthesis and inhibition of degradation; the cells accumulated mannitol, leading to osmoregulation. No significant elongation of* V. volvacea* fruiting bodies during storage at 4°C was observed, and these organs tended to shrink and collapse. The sensory quality of mannitol-treated fruiting bodies was much better than that of control fruiting bodies. Application of a mannitol solution at the cultivation stage of* V. volvacea* somewhat improved the low-temperature resistance of the fruiting bodies, verifying the correlation between mannitol and resistance to this stress in* V. volvacea*. The results of this study lay a foundation for a deeper understanding of the autolysis mechanism of* V. volvacea*, providing technical support for increasing the cryopreservation time of this species and extending the postharvest shelf life of its fruiting bodies. In addition, the mechanism underlying the low-temperature tolerance of the VH3 strain should be further explained at the molecular level.

## 1. Introduction

The fungus* Volvariella volvacea* (*V. volvacea* Bull. ex Fr.) Sing. is an edible mushroom with a unique taste and high nutrient content and is internationally recognized as “a very good source of protein” [1, 2].* V. volvacea *is a thermophilic mushroom and is consequently sensitive to low temperature, and* V. volvacea* mycelial growth is significantly inhibited at temperatures lower than 10°C. Furthermore, at the conventional storage temperature of 4°C,* V. volvacea *mycelia die due to autolysis, and the subentities of this mushroom become soft, decompose, lose vitality, and even die [3–5]. Cultivation and storage of this species require a strict ambient temperature, thus restricting the development of the* V. volvacea* industry. Overall, autolysis is the characteristic that makes this species unsuitable for low-temperature preservation. Although the low-temperature autolysis of* V*.* volvacea* has been studied previously, the underlying mechanism of action has not been clearly described [3].

Mannitol, so named because it was first isolated from the manna ash tree by Proust in 1806 [6], is one of the most important polyols in fungal cells. Mannitol is found in ascomycetes and basidiomycetes, in the hyphae of adelomycetes and zygomycetes, and in sporophores and spores [7, 8]. Mannitol has important biological functions under conditions of stress, such as regulation of osmotic pressure and removal of ROS. For example, Weinstein et al. [9, 10] found that mannitol has a protective effect against low-temperature stress in* Humicola marvinii.* Under hyperosmotic stress conditions, the intracellular mannitol content of* Aspergillus nidulans* changes significantly [11]. Additionally, mannitol is the most abundant polyol under conditions of low osmotic stress in* Uromyces fabae*, which also exhibits arabinitol accumulation under hyperosmotic conditions [12].* Agaricus bisporus* gradually accumulates mannitol under high-salt stress, and the expression level of mannitol dehydrogenase also gradually increases [13]. Nonetheless, mannitol does not play a protective role against adversity in all fungi. For example, in* Stagonospora nodorum*, knockout of mannitol-1-phosphate dehydrogenase (*MP*) and NADP-dependent mannitol dehydrogenase (*MD*) does not affect normal growth of the mutants in hyperosmotic environments, suggesting that mannitol is not involved in osmotic pressure regulation in this species [14]. Overall, mannitol has become a widely studied osmotic regulatory substance in both plants and fungi in recent years [8, 15–19]. However, the action of mannitol in the low-temperature response of the edible fungus* V. volvacea* has not yet been reported. Therefore, based on our previous analysis of the low-temperature transcriptome of* V. volvacea*, the present study aims to explore the mechanism of the protective effect of mannitol on* V. volvacea* mycelia at low temperature and to quantitatively analyze expression of genes encoding enzymes associated with mannitol and relevant metabolic pathways. In addition, this study is the first to add mannitol during cultivation of* V. volvacea* to explore the low-temperature resistance of fruiting bodies from the perspective of cultivation and to further reveal the mechanism of mannitol in protection of* V. volvacea* from low-temperature autolysis. The results provide ideas for further scientific research and a theoretical basis for cultivation of low-temperature-resistant* V. volvacea*.

## 2. Materials and Methods

### 2.1. Testing Strains and Collection of* V. volvacea* Mycelia

*V. volvacea* strain V23 adopted in this study is commonly used in production and is sensitive to low temperature. The VH3 strain was derived by mutagenesis of V23 to generate low-temperature resistance. Both strains were provided by the Culture Collection Center of the Institute of Edible Fungi at the Shanghai Academy of Agricultural Sciences.

Strains V23 and VH3 were placed on solid culture medium and incubated for 4 d at a stable temperature of 32°C. Then, the strains were moved to a flask with liquid medium and incubated at 32°C and 150 rpm for 5 d.

After hyphal growth, the flask was placed in an ice bath at 0°C for 2, 4, 6, or 8 h. The blank control group was treated at 0°C for 0 h. The aseptically cultured, sterile-filtered mycelia were washed several times with sterile water, dried on sterile absorbent paper, and gathered in a sterile centrifuge tube. After freezing in liquid nitrogen, the mycelia were stored at −80°C.

### 2.2. Extraction of Mannitol and Quantitative Measurement

For the determination of mannitol content, mycelia were freeze-dried and ground into powder by a TissueLyser (Qiagen, Hilden, Germany). The method for determining mannitol levels in* V. volvacea* mycelia by high-performance anion chromatography-pulsed amperometric detection (HAPEC-PAD) has already been described [20, 21].

The samples (0.1 g) were ultrasonically extracted in 10 mL of ultrapure water for 30 min and centrifuged at 3600 × g for 20 min at temperature of less than 25°C. Then, the supernatant was filtered with a 0.45-*μ*m filter (Millipore, Bedford, MA, USA) and diluted 5 times for analysis by an ICS2500 HPAEC-PAD system comprising a GP50 quaternary gradient pump, an EG50 automatic eluent generator, an LC30 column oven, a Dionex CarboPac MA1 column, and an ED50 electrochemical detector (Dionex, Sunnyvale, CA, USA). The temperature of the column was 30°C, the mobile phase was 480 mM NaOH solution, and the flow rate was 6.67 *μ*L s^−1^. The adopted external standard mixture included mannitol (Sigma, USA). Each standard was dissolved in deionized water and diluted to several standard solutions to generate a calibration curve [22].

### 2.3. Extraction of* V. volvacea* Mycelial RNA and Reverse Transcription

TRIzol (Tiangen, Beijing, China) was used to extract the total RNA from* V. volvacea* mycelia based on the manufacturer's instructions. The RNA was then dissolved in 50 *μ*L of water (pretreated with DEPC) and analyzed by electrophoresis on a 1% agarose gel. After eliminating genomic DNA, the RNA was reverse transcribed to cDNA following the instructions of the reverse transcription kit (Prime Script RT Reagent Kit with gDNA Eraser). The cDNA was then used as the template for reverse transcription quantitative PCR (RT-qPCR) and stored at −20°C until the next use.

### 2.4. Regions for Coding of Target Genes and the Design and Synthesis of Quantitative PCR Primers

Gene sequences of coding regions were obtained from NCBI based on the expression profiles and gene accession numbers of species homologous to* V. volvacea *and aligned to the whole-genome sequence of* V. volvacea* (https://www.ncbi.nlm.nih.gov/). In GenBank, DNA and eukaryotic protein sequences were compared by BlastX to eliminate potential introns (GT-AG) and obtain the coding sequences of the target genes. Based on the high copy numbers and stability of the internal standard genes of* V. volvacea* at different temperatures, tubulin alpha (*TUB*) was used as the internal standard for quantifying target genes [23]. Primers were designed by using Primer 5.0 software and were typically between 17 and 24 bp.

### 2.5. Amplifying the Target Fragment and Preparing the Standard Plasmid

Using conventional PCR, the target fragment was amplified, and the desired product was detected by electrophoresis on a 1% agarose gel. After purification, the PCR product was ligated to the pGEM®-T vector (Promega, Shanghai, China) to build a standard plasmid and produce a standard curve for RT-qPCR [24].

### 2.6. Fluorescent Quantitation of Genes

A mixture of the above plasmid diluted with target and internal standard genes was used as the template for RT-qPCR amplification according to the quantitative relative ΔΔC_T_ method to construct a standard curve. cDNA obtained from* V. volvacea* mycelia incubated at 0°C for different time periods was used as the template, and quantitative amplification of the internal standard and target genes was performed. Each sample was examined in three replicates using ddH_2_O as the blank control (the system of the amplification reaction is described in Table 1).

The reaction conditions were as follows: 95°C for 20 s; 40 cycles of 95°C for 5 s, 60°C for 15 s, and 72°C for 15 s. The reaction mixture was prepared in an ice bath.

### 2.7. Exogenous Mannitol Spray Treatment

As the culture materials for the fruiting test, cottonseed hulls and lime were weighed according to a mass ratio of 95% to 5%, respectively; the pH value was 10. The cultivation process of* V. volvacea* was carried out as usual.

Mannitol treatment group (M): Fruiting bodies were sprayed with 10% mannitol solution; each basket was sprayed with approximately 100 mL. At 6 d of treatment, water was added once.

Blank control group (CK): Fruiting bodies were sprayed with water; each basket was sprayed with approximately 100 mL. At 6 d of treatment, water was added once.

### 2.8. Storage after Harvest

Egg-shaped* V. volvacea* with an intact umbrella was identified before harvesting. The fruiting bodies were selected and placed in a plastic tissue culture flask with a diameter of 9 cm and a height of 8 cm. Kraft paper was used to seal the flask, which was then stored at 4°C at 80% humidity for 0, 6, 12, 18, 24, 30, 36, 42, and 48 h.

### 2.9. Index Determination and Analysis of Data

#### 2.9.1. Determination of Sensory Index

Sensory index: The sensory evaluation was conducted by a digital scoring method based on the indicators listed in Table 2. The final score was the sum of the scores of each index.

#### 2.9.2. Determination of Weight Loss Rate

Weight loss rate = (fresh weight prior to storage – fresh weight at the end of storage) / fresh weight before storage × 100%

#### 2.9.3. Morphometric Determination

To measure the variation of the morphological tendency of the fruiting bodies in the process of storage, the diameter of the boundary between the white and brown sections in the middle of the fruiting body was taken as the middle diameter. Each index was measured by using vernier calipers. Determinations of each treatment group were performed in 10 replicates.

Reduced middle diameter = (initial middle diameter – middle diameter after storage) / initial middle diameter × 100%

Reduction in length = (initial length – poststorage length) / initial length × 100%

#### 2.9.4. Differences Data Analysis

The extraction and determinations were all carried out in 3 replicates to analyze the data by SPSS (version 11.0) software, with* P* < 0.05 indicating a significant difference. The results are presented as the means ± SD. One-way analysis of variance (ANOVA) with a 95% confidence interval was used to evaluate the significance of the differences between means.

## 3. Results and Discussion

### 3.1. Analysis of Mannitol Metabolic Pathway-Related Genes

**Figure 1:**
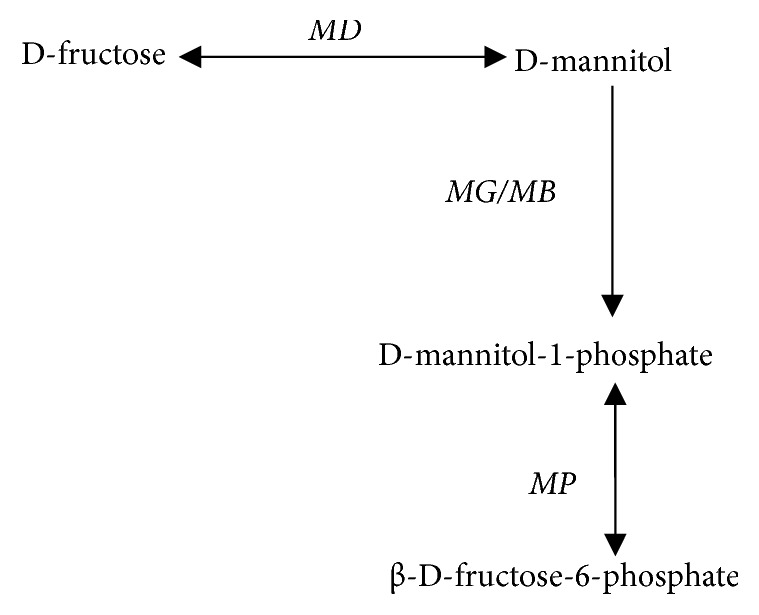
Mannitol metabolic pathway in* V. volvacea.*

Various fungi have different mannitol metabolic pathways, involving a complete cycle between mannitol and fructose, 1-phosphoric acid mannitol and fructose 6-phosphate. However, it remains unknown whether this cycle exists in* V. volvacea*. Therefore, based on the amino acid sequences of enzymes involved in mannitol metabolic pathways in species with homology, BLAST comparison was performed using the entire genome of* V. volvacea*. The putative mannitol metabolic pathway obtained is shown in Figure 1. Synthesis of mannitol in* V. volvacea* is associated with the fructose conversion pathway of* MD*, and the degradation of mannitol occurs via transphosphorylation to generate 1-phosphate mannitol, which is then dehydrogenated to obtain *β*-D-fructose-6-phosphate.

**Figure 2:**
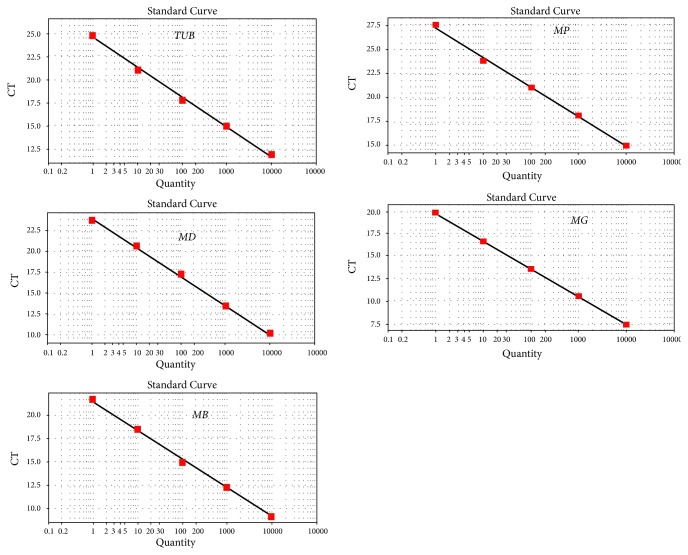
The standard curve of the target genes.

**Figure 3:**
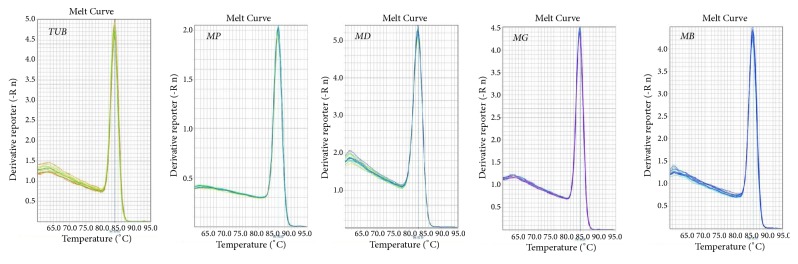
Melting curves of the internal standard gene and target genes. The single peak of the melting curve of the internal standard gene and the target gene confirmed the strong specificity of the amplification and, in turn, the reliability of the results.

The quality of the total RNA obtained by direct extraction influences the accuracy of RT-qPCR results and the quality of cDNA. Electrophoretic analysis of the extracted total RNA revealed complete and distinct bands, indicating that the RNA was not degraded. When routine amplification by PCR was completed, the length of the band corresponding to the target gene was consistent with the predicted length of the fragment specifically amplified by the primers (Table 3), and the detection of a single band indicated that the primer had enough specificity for amplification by RT-qPCR. Cloning and sequencing (Table 4) demonstrated that the sequence of the amplified target gene fragment was identical to the original sequence. The standard curve of the target gene had an R^2^ value greater than 0.99, indicating a good linear relationship (Figure 2). The standard curve could therefore be used to quantify unknown samples accurately. In addition, the single peaks of the melting curves of the target gene and the internal standard gene (Figure 3) indicated that the amplification had strong specificity and could provide accurate results.

A standard curve can be used to quantify unknown samples accurately. For the target gene, the standard curve R^2^ was greater than 0.99. The slope K value met the condition |ΔK| ≤ 0.1. RT-qPCR was thus conducted using the ΔΔCT method. Three biological replicates were performed.

#### 3.1.1. Analysis of the NADP-Dependent Mannitol Dehydrogenase (*MD*) Gene

**Figure 4:**
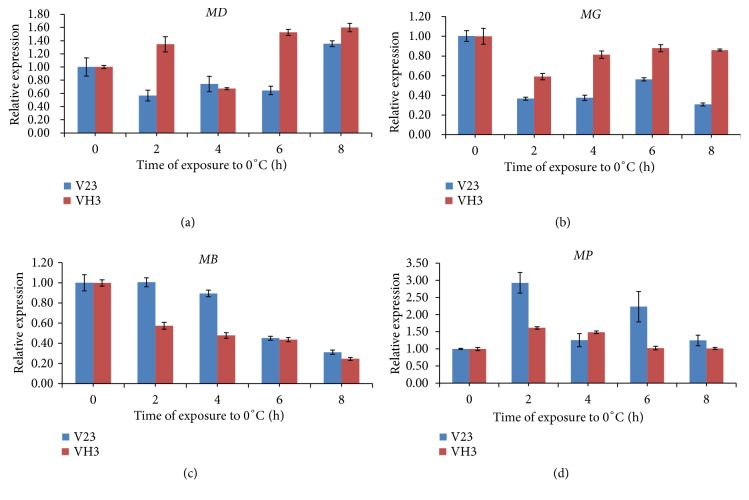
In the V23 and VH3 strains, the relative expression of every gene in the mannitol metabolic pathway was determined. Values shown are the means ± SD of three replicates (n = 3). The vertical bars represent the standard errors of the average value. The letters reveal the expression of each gene, i.e., (a)* MD*, (b)* MG*, (c)* MB*, (d)* MP*.

Mannitol dehydrogenase is an NADH-dependent enzyme that reversibly catalyzes conversion between D-mannitol and D-fructose. Because the degradation pathway of D-mannitol in* V. volvacea* is unidirectional, the results show that catalysis by mannitol dehydrogenase favors mannitol synthesis. Based on quantitative results for* MD* gene expression (Figure 4(a)), levels in the V23 strain, which is not tolerant to low-temperature treatment, exhibited a decreasing trend after cells were subjected to low-temperature stress, and this low expression was maintained for 6 h of treatment. The gene expression increased slightly at 8 h, with higher levels than those observed without low-temperature treatment. Despite the increase in the expression level of this gene in VH3 after 2 h of low-temperature treatment, the increase was not significant, and levels decreased at 4 h of treatment. Overall, the expression of the* MD* gene significantly increased, with a high level maintained at 8 h. At all other time points, the expression of the* MD* gene was greater in strain VH3 than in strain V23 under low-temperature treatment, excluding 4 h, when the level was relatively low. It can be seen that, compared to strain V23, strain VH3 more highly expresses this mannitol pathway gene and that synthesis of mannitol is also higher in strain VH3 than in strain V23. Therefore, strain VH3 could exhibit better resistance to low temperature than strain V23.

#### 3.1.2. Analysis of the Glycosyltransferase Family 1 Protein (*MG*) and ATP Phosphoribosyltransferase (*MB*) Genes

Ribosyltransferases and transphosphorylation ribosylases are two types of enzymes that transfer phosphoric acid in the intracellular phosphotransferase system (PTS). In addition to catalyzing conversion of D-mannitol to D-mannitol-1-phosphate via transphosphorylation, these enzymes are important in the catalysis of other sugars and sugar alcohols in PTS. Thus, it is hypothesized that under low-temperature conditions, changes in the expression levels of the ribosyltransferase (*MG*) gene and transphosphorylation ribosylase (*MB*) gene in* V. volvacea* are relevant to mannitol metabolic pathway regulation. Changes in expression are also affected by the regulation of other glucose metabolic pathways. After low-temperature stress, decreasing trends in the overall expression levels of both* MG* (Figure 4(b)) and* MB* (Figure 4(c)) were observed. Expression of the* MG* gene began to increase after 4 h, suggesting regulation of the physiological system involved in ribose transfer, and VH3 strain showed a higher level of expression than the V23 strain. After stimulation by low temperature,* MB* gene expression levels in the mycelia of* V. volvacea* showed an overall decreasing trend, and the level was not restored with prolonged treatment. These findings indicate that the metabolic pathway involved in transphosphorylation of ribosylase is not directly related to the response mechanism to cold stress. As cells were severely damaged at low temperature, and the expression level of the gene encoding the enzyme also decreased.

#### 3.1.3. Analysis of the Mannitol-1-Phosphate Dehydrogenase (MP) Gene

*MP* catalyzes the dehydrogenation reaction in the mannitol catabolism pathway, resulting in the conversion of D-mannitol-1-phosphate to *β*-D-fructose-6-phosphate followed by *β*-D-fructose-6-phosphate, which enters into other carbon-cycle pathways. Quantitative results of* MP* gene expression levels (Figure 4(d)) show an upward trend in the mycelia of* V. volvacea* at 4 h of low-temperature stress. A decrease in expression was found after 4 h of treatment, after which the level remained steady. In the V23 strain subjected to low temperature, expression of the* MP* gene increased at 2 h, decreased at 4 h, increased again significantly at 6 h, and decreased to the level observed in untreated mycelia at 8 h. The reason is that at the initial stage of low-temperature stimulation, mannitol was the most abundant energy storage material in the V23 strain and was heavily degraded, with the energy being used to maintain physiological metabolism in response to low temperature. However, osmotic adjustment of the levels occurred after 4 h of treatment; the degree of mannitol degradation decreased and then started to accumulate in the mycelia. The expression level of the* MP *gene, which encodes the enzyme that catalyzes decomposition, increased in the VH3 strain in the initial phase of low-temperature stimulation and began to decrease again at 4 h of treatment, maintaining this low level. The VH3 strain showed a lower expression level of* MP* than the V23 strain at all time points except 4 h. It appears that the VH3 strain, with reduced tolerance to low temperature, initiated osmotic adjustment after 4 h of treatment; the expression levels of the enzymes that catalyze degradation of mannitol exhibited a decreasing trend and remained at levels similar to those in untreated mycelia.

### 3.2. Analysis of the Relationship between Mannitol Metabolic Pathways and the Low-Temperature Resistance of* V. volvacea*

When mycelia of strains V23 and VH3 were subjected to low-temperature treatment, the expression conditions of the enzymes in mannitol metabolism were altered to different degrees. Differences in the expression levels of the genes encoding various enzymes were observed at different times during low-temperature treatment, indicating that the metabolic response of mannitol to low temperature differs in the two strains. It is hypothesized that the changes and differences in expression of genes encoding these enzymes are associated with a mannitol-based mechanism of low-temperature resistance in* V. volvacea* mycelia.

During the first 2 h of low-temperature stress, the levels of the* MP* gene increased significantly. The results suggest that, when stimulated by low temperature, the reaction product of the mannitol degradation pathway catalyzed by these enzymes was produced in large amounts and entered the carbon cycle to resist the stress and provide energy. With prolonged treatment, the expression level of the* MP* gene exhibited a decreasing trend, indicating that the decomposition of mannitol decreased and intracellular accumulation of mannitol occurred due to osmotic regulation induced by the stress, leading to the mannitol osmoregulatory effect. The expression levels of* MG*, encoding glycosyltransferase, decreased at the initial stage of low-temperature stimulation and increased with prolonged treatment, indicating that the enzyme not only catalyzes the decomposition of mannitol but also participates in other physiological pathways involved in the transfer of phosphate groups. Therefore, when cellular metabolism was inhibited by low-temperature stress, the expression level of this gene was also reduced and then increased as the low-temperature stress mechanism of resistance was initiated. Accordingly, the VH3 strain showed higher expression levels than the V23 strain.

Expression of the* MD* gene encoding mannitol dehydrogenase, which is involved in mannitol synthesis in* V. volvacea* mycelia, first exhibited a decreasing trend and then an increasing trend during low-temperature treatment, suggesting that synthesis of mannitol was inhibited at the initial stage of treatment. At 6 h, the low-temperature resistance mechanism was invoked, and* MD* expression increased. These findings suggest that high levels of mannitol synthesis were produced for osmoregulation. In summary, at the initial stage of low-temperature treatment, intracellular mannitol was largely catabolized as an energy storage material, and the expression levels of genes encoding enzymes in physiological responses were also reduced. After a period of treatment, the cells initiated a low-temperature resistance mechanism, the mannitol synthesis reaction was activated, and the degradation reaction was inhibited, followed by mannitol accumulation for osmoregulation.

### 3.3. Changes in Intracellular Mannitol Levels at Different Time Points of Low-Temperature Treatment

**Figure 5:**
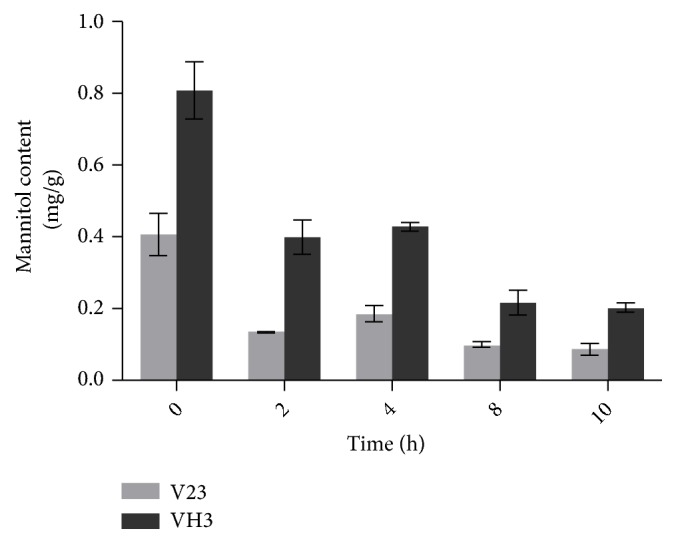
Changes in mannitol content in the mycelia of* V. volvacea* cultivated at 0°C for 0, 2, 4, 8, and 10 h. The data are presented as the means ± SD from three independent replicates (n = 3).

To further examine the effect of mannitol in* V. volvacea* under low-temperature stress, mannitol contents were determined by HAPEC-PAD. As shown in Figure 5, in strain VH3, the content of mannitol was higher than in strain V23. When subjected to low temperature, the content of mannitol notably decreased in the first 2 h and then increased slightly after 4 h. However, as the duration of stress continued, the content of mannitol decreased again and tended to be stable. During the entire process of stress treatment, both showed a downward trend; nonetheless, the content of mannitol in the VH3 strain was always higher than that in the V23 strain, which may explain why the VH3 strain is relatively resistant to low temperature. It can be speculated that within the initial 2 h, low temperature caused decomposition of a large amount of intracellular mannitol. After 4 h,* V. volvacea* may have initiated low-temperature regulation measures and regulation of infiltration, resulting in an increase in mannitol content. The overall response to low-temperature stress is essentially consistent with the expression profile of genes related to the metabolic pathway of mannitol, and the possible reason for the individual differences is that there are alternative pathways for mannitol metabolism in* V. volvacea*. There are many hypotheses to date regarding mannitol metabolic pathways in fungi [14, 19]. Hunt et al. first reported the metabolic cycle of mannitol in a study of polyketide formation in* Alternaria alternata* hyphae [25]. This cycle is a branch of the glycolytic pathway in which fructose-6-phosphate is reduced to mannitol 1-phosphate through 1-phosphate mannitol dehydrogenase, followed by decomposition to mannitol under the action of mannitol-1-phosphatase. Therefore, mannitol is oxidized to fructose, which is activated by hexokinase and catalytically converted to fructose-6-phosphate, constituting a complete closed cycle [26]. Mannitol dehydrogenase and mannitol-1-phosphate dehydrogenase have been isolated and purified from ascomycetes, basidiomycetes, and deuteromycetes [27]. However, when mannitol-1-phosphate was detected in cells, the activities of these two enzymes were not necessarily detected at the same time. For example, mannitol-1-phosphate is present in shiitake mushrooms, but only the activity of mannitol dehydrogenase was detected [28, 29]. By contrast, only mannitol-1-phosphate dehydrogenase activity was detected in a mycelial extract of* Penicillium notatum*. Another cyclical pathway of mannitol has been suggested for zygomycetes [30]. Additionally, a new mannitol dehydrogenase can catalyze a reversible reaction to generate fructose, which is converted back to fructose-6-phosphate to enter the pathway of glycolysis. Mannitol kinase can convert mannitol to mannitol-1-phosphate by adding a phosphate group [30], although this pathway has been verified only in some zygomycetes. Overall, mannitol kinase activity remains to be further studied [15]. In* Stagonospora nodorum*,* MD* and* MP* have been knocked out as single or multiple genes. The phenotype of the* △MD* mutant is almost identical to that of the wild-type strain; although mannitol production was not affected, growth was slightly inhibited on media where mannitol was the sole source of carbon [31].* MP* gene knockout in* Aspergillus niger* reduced the mannitol content by 70%; although mannitol synthesis in mycelia did not occur, decomposition and utilization of mannitol were not affected [32]. When* Aspergillus* and* Sphaerosporella brunnea* were grown on mannitol-containing media, intracellular* MTD* and* MPP* activity decreased, and* MPD* activity increased [33]. These studies demonstrate that mannitol-1-phosphate dehydrogenase plays an important role in mannitol decomposition. A recent study found that with simultaneous* MD* and* MP* gene knockout in* Botrytis cinerea*, the mutant strains continued to accumulate mannitol, indicating the existence of a new mannitol synthesis pathway in this fungus [34]. The classic hypothetical pathway of cyclical mannitol metabolism is controversial. It is believed that new mannitol metabolic pathways may be discovered with further research.

### 3.4. Influence of Exogenous Mannitol on the Resistance of* V. volvacea* Fruiting Bodies to Low Temperature

The above results showed that the intracellular mannitol could activate the emergency regulation mechanism to respond to the low-temperature stress when* V. volvacea* was subjected to it. In order to further verify the protective effect of mannitol on* V. volvacea* under low-temperature stress, we studied the response of fruiting body to low temperature by adding exogenous mannitol. This study is the first to apply a mannitol solution to the fruiting bodies of* V. volvacea* in an attempt to improve resistance to low temperature.

**Figure 6:**
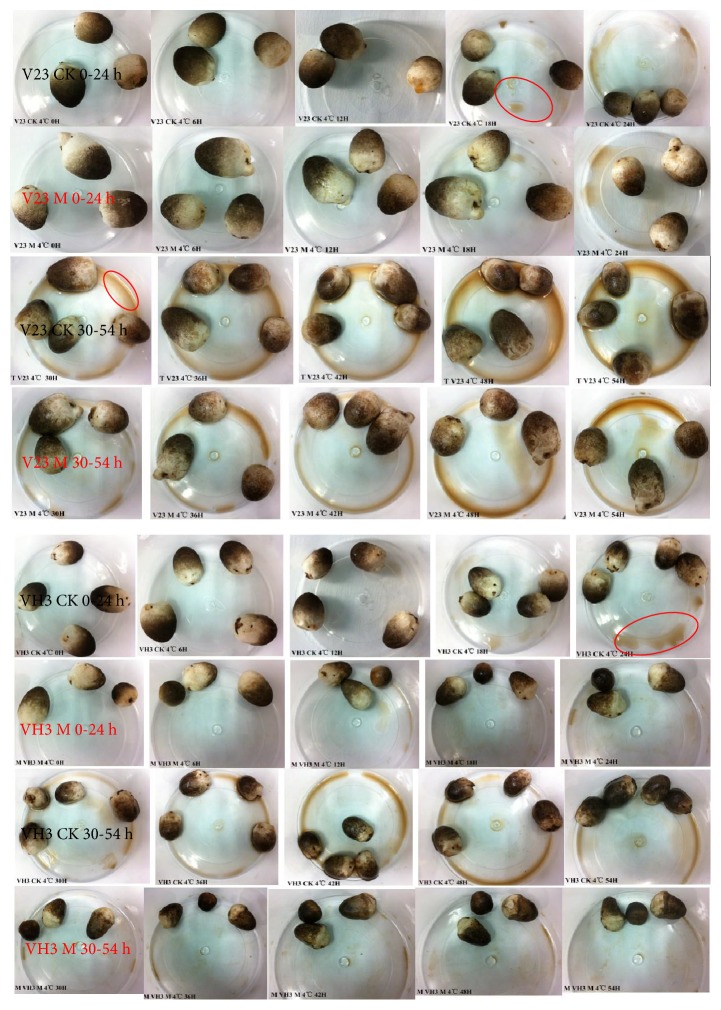
Sensory properties of* V. volvacea* fruiting bodies at 4°C with different treatments. Images were obtained at 6 h intervals, and the morphological variation of the two strains at 4°C was assessed. M indicates the group treated with mannitol; CK indicates the control group.

**Figure 7:**
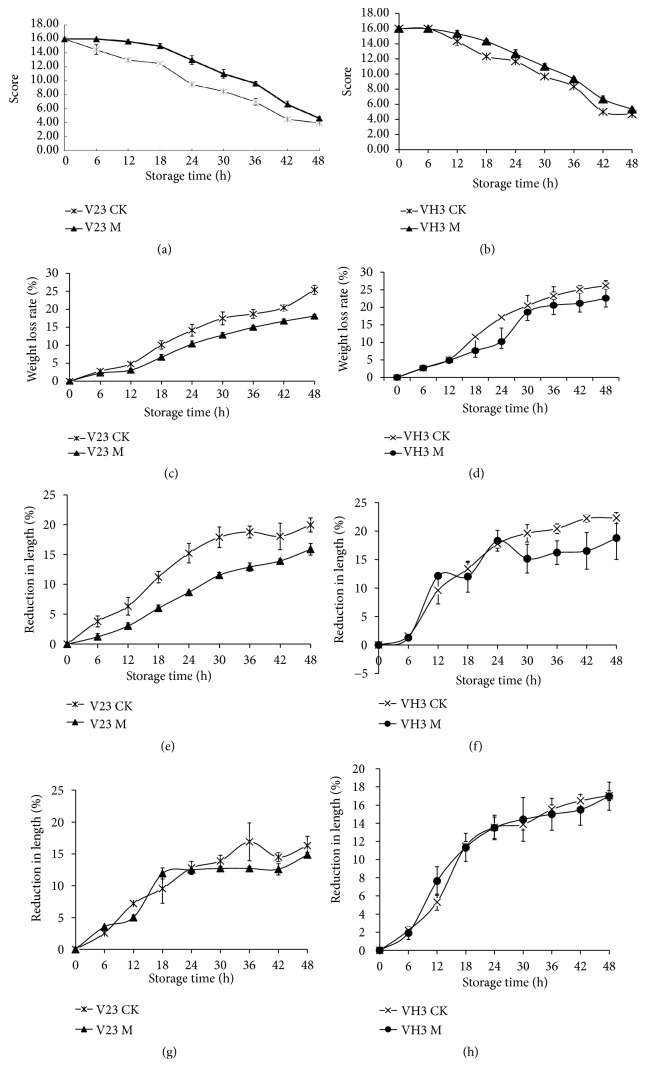
Sensory indexes of the V23 and VH3 strains: mannitol treatment group (M) and blank control group (CK). Tendency chart of sensory properties (a, b); weight loss rate (c, d); middle diameter reduction rate (e, f); length reduction rate (g, h). Values are the means of standard errors (n = 3). The vertical bars represent the standard errors of the average.

Under different treatment conditions, the sensory properties of the* V. volvacea* fruiting bodies at 4°C are shown in Figure 6. According to the chart showing trends in sensory quality (Figures 7(a) and 7(b)), overall scores of hardness, flavor, color, and wetness of* V. volvacea* fruiting bodies exhibited decreasing trends with prolonged storage time at a low temperature of 4°C. The overall sensory quality scores of the untreated blank group of* V. volvacea* fruiting bodies were obviously lower than those of the group treated with mannitol solution and stored at 4°C. As for the blank control group, the overall sensory index of the V23 strain, which is sensitive to low temperature, was lower than that of the VH3 strain, and there was no significant difference in the sensory quality of the mannitol-treated group. Overall, the sensory quality scores indicate that mannitol addition has a positive effect on the low-temperature resistance of fruiting bodies in the process of storage.

As shown in Figures 7(c) and 7(d), the weight loss rate of the fruiting bodies of* V. volvacea* increased with prolonged storage at 4°C, and the mannitol-treated group had a significantly lower value than the blank control group. The result showed that the addition of mannitol correlates with reduced weight loss of* V. volvacea* fruiting bodies during storage at low temperature.

In addition, middle diameter reduction rate data (Figures 7(e) and 7(f)) showed an increasing trend for fruiting bodies of* V. volvacea* with prolonged storage at 4°C, which indicates that the fruiting bodies shrank after water loss and collapsed in the middle. For the reduction rate of the middle diameters, the treated V23 strain fruiting bodies showed a significantly lower reduction rate than the blank control group. The same result was observed for VH3. Thus, exogenous mannitol was correlated with a decrease in the middle diameter reduction rate during storage at 4°C.

Moreover, as presented in Figures 7(g) and 7(h), the reduction rate of fruiting bodies exhibited an increasing trend with prolonged storage at 4°C. However, the effect of mannitol treatment on reducing the fruiting body diameter during storage was not significant. According to these results, the length of* V. volvacea *fruiting bodies remains stable during storage, with no significant elongation.

In summary,* V. volvacea* was sprayed with water during the cultivation process, and the osmoregulatory substance mannitol was added during the water application step; fruiting bodies were harvested at 4°C. The data regarding sensory traits at low temperature revealed higher indicators in the treatment group than in the blank control group. The main indicators of fruiting body autolysis at low temperature include softening, liquefaction, and odor. The weight loss rate measures the liquefaction level; the bottom diameter reduction rate, the middle diameter reduction rate, and the mushroom length reduction rate measure the level of fruiting body softening. Data analysis showed that the fruiting bodies did not significantly elongate during storage but tended to shrink and collapse; however, the sensory quality of the fruiting bodies showed a lower value in the treatment group than in the blank control group. The results show that mannitol treatment has a stimulative effect on the resistance of* V. volvacea* fruiting bodies at 4°C. This conclusion is also consistent with the protective effect of mannitol on* V. volvacea* mycelia at low temperature, as reported by Chen Mingjie et al. [35].

## 4. Conclusion

In this study, the role of mannitol metabolic pathway-associated genes and changes in intracellular mannitol contents and exogenous mannitol in the cold stress response of* V. volvacea* were assessed to show that mannitol affects the low-temperature resistance of* V. volvacea*. When subjected to low-temperature stress, synthesis of mannitol was inhibited at the initial stage, after which synthesis of mannitol was activated with prolonged exposure, and the cells tended to synthesize large amounts of mannitol for osmoregulation. However, other mannitol synthesis pathways in* V*.* volvacea* require further examination. Application of a mannitol solution at the cultivation stage of* V. volvacea* improved to a certain extent the low-temperature resistance of the fruiting bodies, verifying the correlation between mannitol and resistance to this stress in* V. volvacea*. The results of this study lay a foundation for a deeper understanding of the autolysis mechanism of* V. volvacea*, providing technical support for increasing the cryopreservation time of this species and extending the postharvest shelf life of its fruiting bodies. In addition, the mechanism of the low-temperature tolerance of the VH3 strain should be further explained at the molecular level.

## Figures and Tables

**Table 1:** RT-qPCR reaction system.

Reagent	Volume
SYBR Premix Ex TaqTM (2×)	10 *μ*L
Forward primer (10 *μ*mo1 L^−1^)	0.4 *μ*L
Reverse primer (10 *μ*mo1 L^−1^)	0.4 *μ*L
Rox	0.4 *μ*L
ddH2O	6.8 *μ*L
cDNA	2 *μ*L
Total	20 *μ*L

**Table 2:** Criteria for sensory evaluation.

Sensory index	Scores			
	4	3	2	1
Hardness	Texture is full Hard	Slightly inelastic Hardness is acceptable	Soft texture	Soft texture Not edible
Flavor	Odorless Typical mushroom flavor	Fresh mushroom flavor Slight odor Market is acceptable	Slightly fresh Mushrooms have obvious smell Limited application	No fresh mushroom flavors Serious odor Not edible
Color	No change	Small areas of browning	Browning of most areas of the mushroom	Browning of the whole mushroom
Water condition	None The surface is not sticky	Small amount of water The surface is slightly sticky	Water The surface is sticky	Large amount of water The surface is very sticky

**Table 3:** Primers for RT-qPCR.

Primer	Base number	Gene sequence
*MP-F*	20	5′-CCATCCCCAAAGTCCAAAGA-3′
*MP-R*	24	5′-CCTGTGCAGAATAGCTTTACGAGA-3′
*MD-F*	22	5′-TCATCAACCAGGCAGGACTCAA-3′
*MD-R*	22	5′-GTTATTCACCGCAGCCTTGGAC-3′
*MG-F*	17	5′-TCCCGAACCAGCGAGAA-3′
*MG-R*	22	5′-GAAGAAGCGGATAAGGAACGAA-3′
*MB-F*	17	5′-CGCCGAAACGCTTGATG-3′
*MB-R*	20	5′-ACGAGTGCGATAGGGTGGTT-3′

**Table 4:** Results of cloning and sequencing.

Gene	Cloning and sequencing results	Sequence length
*TUB*	GATTACCACCTGTTACTTGCCCCGAGGATGATCCGTGAACT	116
	CATCCCTGAAGCTGTCCTCGGCTTGTTCGTGCACACACCAT	
	TCCCAAGCAGCGAAGTTTTCCGATGCCTACCTCGCCGCAAG	
	GAGATACTCGATGGCATGCTAGGCGCCAACCTTGTTTGCTT	
	CCAAACATACTCCTACGCTCGTC	
*MP*	CCATCCCCAAAGTCCAAAGAGCTGCAGTTGTGTCCTCAGCT	133
	GGAGCTCCTATCGAAATTCGCCACGATGTACCAGTCAAGCA	
	AGCGTCCGAGCTCGCCGCTGGGGAGTGTCTCGTAAAGCTAT	
	TCTGCACAGG	
*MD*	TCATCAACCAGGCAGGACTCAACCGCCCTCTCACTCAGCAA	86
	CCGCTGACAGTGTTCTACAACTCGTCCAAGGCTGCGGTGAA	
	TAAC	
*MG*	TCCCGAACCAGCGAGAAGACTAGGGATTCATCTACGAAAC	140
	GCACTAGCTTGGTAGCCGCAGTCTGGTCCGTCCTACCTGAT	
	GCTTTTACTTCAACAGGTTCCCCCAAGAATTCACGCCTTCGT	
	TCCTTATCCGCTTCTTC	
*MB*	CGCCGAAACGCTTGATGGCCGCCTATTGTTCGCGATCCCTA	156
	AAAAAGGGAGATTGCATGAAAAGTGCCTGTCGCTGCTCGC	
	AGGCGCCGATATACAGTTCCGAAGGCACAACCGCTTAGAT	
	GTGGCCCTTGTATTAAACCACCCTATCGCACTCGT	

## Data Availability

The dataset supporting the conclusions of this study is available and we have agreed to share the dataset. You can contact us by email to obtain the raw data in our manuscript.

## Abbreviations

*V. volvacea*: * Volvariella volvacea*
RT-qPCR: Reverse transcription quantitative PCR
HAPEC-PAD: High-performance anion chromatography-pulsed amperometric detection
*MD*: NADP-dependent mannitol dehydrogenase
*MG*: Glycosyltransferase family 1 protein
*MB*: ATP phosphoribosyltransferase
*MP*: Mannitol-1-phosphate dehydrogenase
CK: Blank control group
M: Mannitol treatment group
ANOVA: One-way analysis of variance.

## References

[1] Orilto C. A., Carangal A. (1961). Nitrogenous constituents of *volvariella volvacea*. *Philippine Agricultural Scientist*.

[2] Luo G. L. (1995). The nutritional value of *V. volvacea*. *Sichuan Food and Fermentation*.

[3] Chang S. T., Hayes W. A. (1978). *The Biology and Cultivation of Edible Mushrooms*.

[4] Wang F. M., Gong X. R., Gao J. H., Liu R. X. (1990). Study on preservation of *V. volvacea* after harvest. *Edible Mushrooms*.

[5] Kerrigan R. W., Chang S., Buswell J. A., Chiu S. (1994). Mushroom biology and mushroom products. *Mycologia*.

[6] Wright L. W. (1994). Sorbitol and mannitol. *Chemtech*.

[7] Zhen J. G. (2005). *Functional Sugar Alcohol*.

[8] Patel T. K., Williamson J. D. (2016). Mannitol in plants, fungi, and plant–fungal interactions. *Trends in Plant Science*.

[9] Weinstein R. N., Palm M. E., Johnstone K., Wynn-Williams D. D. (1997). Ecological and physiological characterization of Humicola marvinii, a new psychrophilic fungus from fellfield soils in the maritime Antarctic. *Mycologia*.

[10] Weinstein R. N., Montiel P. O., Johnstone K. (2000). Influence of growth temperature on lipid and soluble carbohydrate synthesis by fungi isolated from fellfield soil in the maritime Antarctic. *Mycologia*.

[11] Beever R. E., Laracy E. P. (1986). Osmotic adjustment in the filamentous fungus aspergillus nidulans. *Journal of Bacteriology*.

[12] Clark A. J., Blissett K. J., Oliver R. P. (2003). Investigating the role of polyols in Cladosporium fulvum during growth under hyper-osmotic stress and in planta. *Planta*.

[13] Stoop J. M. H., Mooibroek H. (1998). Cloning and characterization of NADP-mannitol dehydrogenase cDNA from the button mushroom, Agaricus bisporus, and its expression in response to NaCl stress. *Applied and Environmental Microbiology*.

[14] Solomon P. S., Waters O. D. C., Jörgens C. I. (2006). Mannitol is required for asexual sporulation in the wheat pathogen Stagonospora nodorum (glume blotch). *Biochemical Journal*.

[15] Boonsaeng V., Sullivan P. A., Shepherd M. G. (1976). Mannitol production in fungi during glucose catabolism. *Canadian Journal of Microbiology*.

[16] Tarczynski M. C., Jensen R. G., Bohnert H. J. (1993). Stress protection of transgenic tobacco by production of the osmolyte mannitol. *Science*.

[17] Martin B., Cushman J. C. (2003). Tolerance of mannitol-accumulating transgenic wheat to water stress and salinity. *Plant Physiology*.

[18] Chakraborty T. K., Basu D., Das N., Sengupta S., Mukherjee M. (2004). The mannitol cycle in Pleurotus ostreatus (Florida). *FEMS Microbiology Letters*.

[19] Vélëz H., Glassbrook N. J., Daub M. E. (2007). Mannitol metabolism in the phytopathogenic fungus Alternaria alternata. *Fungal Genetics and Biology*.

[20] Zhou S., Xue J., Liu Y. (2011). Determination of arabitol,trehalose and mannitol in the fruit bodies of edible fungi using high performance anion chromatography-pulsed amperometric detection(HAPEC-PAD). *Acta Edulis Fungi*.

[21] Zhou S., Ma F., Zhang X., Zhang J. (2016). Carbohydrate changes during growth and fruiting in Pleurotus ostreatus. *Fungal Biology*.

[22] Zhou S., Tang Q.-J., Luo X. (2012). Determination of carbohydrates by high performance anion chromatography-pulsed amperometric detection in mushrooms. *International Journal of Medicinal Mushrooms*.

[23] Zhao X., Song X., Li Y. (2018). Gene expression related to trehalose metabolism and its effect on Volvariella volvacea under low temperature stress. *Scientific Reports*.

[24] Wang H., Chen M. J. (2007). The establishment of the standard quality granule and standard curve of Cor3 gene in real-time fluorescence quantitative PCR. *Acta Edulis Fungi | Acta Edulis Fungi*.

[25] Hult K., Gatenbeck S. (1978). Production of NADPH in the mannitol cycle and its relation to polyketide formation in alternaria alternata. *European Journal of Biochemistry*.

[26] Suvarna K., Bartiss A., Wong B. (2000). Mannitol-1-phosphate dehydrogenase from Cryptococcus neoformans is a zinc-containing long-chain alcohol/polyol dehydrogenase. *Microbiology*.

[27] Krahulec S., Armao G. C., Weber H., Klimacek M., Nidetzky B. (2008). Characterization of recombinant Aspergillus fumigatus mannitol-1-phosphate 5-dehydrogenase and its application for the stereoselective synthesis of protio and deuterio forms of d-mannitol 1-phosphate. *Carbohydrate Research*.

[28] Strandberg G. W. (1969). D-mannitol metabolism by Aspergillus candidus.. *Journal of Bacteriology*.

[29] Adomako D., Kaye M. A. G., Lewis D. H. (2010). Carbohydrate metabolism in chaetomium globosum. *New Phytologist*.

[30] Ueng S. T.-H., Hartanowicz P., Lewandoski C., Keller J., Holick M., McGuinness E. T. (1976). D-mannitol dehydrogenase from absidia glauca. purification, metabolic role, and subunit interactions. *Biochemistry*.

[31] Solomon P. S., Tan K.-C., Oliver R. P. (2005). Mannitol 1-phosphate metabolism is required for sporulation in planta of the wheat pathogen Stagonospora nodorum. *Molecular Plant-Microbe Interactions*.

[32] Ruijter G. J. G., Bax M., Patel H. (2003). Mannitol is required for stress tolerance in Aspergillus niger conidiospores. *Eukaryotic Cell*.

[33] Ramstedt M., Jirjis R., Söderhäll K. (2010). Metabolism of mannitol in mycorrhizal and non‐mycorrhizal fungi. *New Phytologist*.

[34] Dulermo T., Rascle C., Billon-Grand G., Gout E., Bligny R., Cotton P. (2010). Novel insights into mannitol metabolism in the fungal plant pathogen Botrytis cinerea. *Biochemical Journal*.

[35] Chen M. J., Song X. X., Guo L. g., Zhao Y., Wang C. G., Yu C. X. (2017). Protective effect of mannitol on volvariella volvacea mycelia at low temperature. *Acta Agriculturae Shanghai*.

